# Real-World Effectiveness and Safety of Tarlatamab in Small Cell Lung Cancer: A Systematic Review

**DOI:** 10.3390/cancers18213550

**Published:** 2026-11-05

**Authors:** Supriya Peshin, Ehab Takrori, Bhavesh Mohan Lal, Konstantinos Arnaoutakis

**Affiliations:** 1 Division of Geriatrics, Department of Internal Medicine, University of Arkansas for Medical Sciences, Little Rock, AR 72205, USA; supriyapeshin@gmail.com; 2 College of Medicine, Alfaisal University, Riyadh 11533, Saudi Arabia; etakrori@gmail.com; 3 Division of Hematology and Medical Oncology, Department of Medicine, Oregon Health & Science University, Portland, OR 97239, USA; bhaveshrathod045@gmail.com; 4 Division of Hematology and Medical Oncology, University of Arkansas for Medical Sciences, Little Rock, AR 72205, USA

**Keywords:** tarlatamab, DLL3, small cell lung cancer, real-world evidence, cytokine release syndrome, ICANS

## Abstract

Background: Tarlatamab, a delta-like ligand 3 (DLL3)-targeted bispecific T-cell engager, has demonstrated clinical efficacy in previously treated small cell lung cancer (SCLC). However, its effectiveness and safety in routine clinical practice, particularly among patients underrepresented in clinical trials, remain incompletely characterized. Objective: To systematically review available real-world evidence regarding the effectiveness and safety of tarlatamab in patients with SCLC. Methods: A systematic review was conducted in accordance with PRISMA 2020 guidelines and registered in PROSPERO (CRD420261345046). PubMed, the Cochrane Library, and ScienceDirect were searched for observational studies reporting real-world effectiveness or safety outcomes. Of 141 identified records, 134 remained after duplicate removal, and five retrospective studies met the eligibility criteria. Study characteristics, effectiveness outcomes, and adverse events were synthesized narratively because of heterogeneity in patient populations, outcome definitions, and follow-up. Results: Five retrospective real-world studies were included. Reported partial or objective response proportions ranged from 35.2% to 66.7% across selected predominantly de novo SCLC cohorts, with estimates frequently restricted to response-evaluable subsets. The EGFR-mutant transformed SCLC cohort was analyzed separately. In one cohort, the reported objective response rate was 66.7% (20/30), corresponding to documented responses in 41.7% (20/48) of all treated patients. Median progression-free survival ranged from 1.7 to 4.9 months across reported analyses. Patients with EGFR-mutant transformed SCLC demonstrated less favorable outcomes, with partial response in 7.1% (1/14) of response-evaluable patients and median overall survival of 2.7 months. Cytokine release syndrome occurred in 54.2–72.7% of patients in selected cohorts, while ICANS occurred in 23.3–47.6%, with substantial variation across study populations and treatment protocols. The overall certainty of evidence was very low. Conclusions: Available real-world evidence suggests that tarlatamab demonstrates clinically meaningful antitumor activity in previously treated SCLC, including patients with adverse baseline characteristics. However, effectiveness estimates are limited by small sample sizes, selection of response-evaluable populations, and heterogeneous outcome assessment. The relatively high incidence of ICANS reported in several real-world cohorts warrants particular attention, although differences in patient characteristics and toxicity ascertainment limit direct comparisons with clinical trials. Larger prospective studies are needed to establish real-world effectiveness and optimize toxicity management. Registration: CRD420261345046.

## Simple Summary

This systematic review evaluated real-world use of tarlatamab in small cell lung cancer (SCLC) and identified only five eligible studies. Overall, tarlatamab showed meaningful antitumor activity in routine practice, including in higher-risk patients often excluded from clinical trials, but CRS and ICANS/neurotoxicity were common and sometimes more frequent than in trials. The evidence remains limited by small, retrospective studies, highlighting the need for larger prospective studies.

## 1. Introduction

Small cell lung cancer (SCLC) is an aggressive malignancy characterized by rapid growth, early dissemination, and frequent relapse despite initial chemosensitivity. Outcomes after progression on platinum-based therapy remain poor, with real-world datasets showing median overall survival (OS) in the range of ~5–6 months following initiation of second-line treatment in contemporary practice [1].

Delta-like ligand 3 (DLL3), an inhibitory Notch pathway ligand, is frequently expressed in SCLC and has emerged as a therapeutically actionable target. Recent translational work highlights DLL3 expression across pulmonary neuroendocrine tumors, including SCLC, supporting the biologic rationale for DLL3-directed strategies while also emphasizing heterogeneity in expression [2].

Tarlatamab is a DLL3-targeted bispecific T-cell engager designed to redirect cytotoxic T cells toward DLL3-expressing tumor cells. Clinical development has demonstrated antitumor activity in previously treated SCLC. In the phase 2 DeLLphi-301 study, tarlatamab produced objective responses with a toxicity profile dominated by immune activation events including cytokine release syndrome (CRS) [3]. More recently, confirmatory randomized evidence in DeLLphi-304 showed that tarlatamab improved OS compared with investigator’s choice chemotherapy among patients whose disease progressed during or after platinum-based chemotherapy [4].

However, translating trial results into routine practice can be challenging for T-cell-redirecting therapies. Toxicities such as CRS and immune-effector-cell-associated neurotoxicity syndrome (ICANS) require structured monitoring, prompt supportive care, and center-specific operational pathways (e.g., inpatient versus outpatient initiation). These factors may influence dose delivery, treatment continuity, and outcomes in real-world populations, which often include patients with poorer performance status, greater comorbidity burden, and central nervous system involvement than those enrolled in clinical trials [5,6,7,8].

At the same time, the tarlatamab literature has expanded rapidly and includes clinical trials, translational/drug-resistance studies, pharmacokinetic and exposure-response modeling, external control/indirect comparisons, and regulatory summaries. While these sources inform efficacy under controlled conditions and support dose selection, they do not directly address how tarlatamab performs in routine clinical settings. Real-world evidence (RWE), including retrospective cohorts and institutional series, can complement trial data by evaluating effectiveness and safety in broader, less-selected populations and by describing implementation outcomes relevant to CRS/ICANS management.

Accordingly, we conducted a systematic review to identify and synthesize published real-world studies reporting effectiveness and safety outcomes of tarlatamab in SCLC, with particular focus on clinically meaningful endpoints (e.g., OS, progression-free survival/time-to-progression, and best response) and immune-mediated toxicity outcomes, including CRS and ICANS.

## 2. Methodology

### 2.1. Protocol and Reporting Framework

This systematic review was designed and reported in accordance with the Preferred Reporting Items for Systematic Reviews and Meta-Analysis (PRISMA) guidance. The review protocol was registered in PROSPERO (CRD420261345046). The literature search was last conducted on 15 May 2026. Accordingly, the findings of this review reflect the real-world evidence available through that date. The review question and eligibility criteria were defined a priori to evaluate real-world and safety outcomes of tarlatamab in patients with SCLC.

### 2.2. Reporting Guidelines

**Figure 1:**
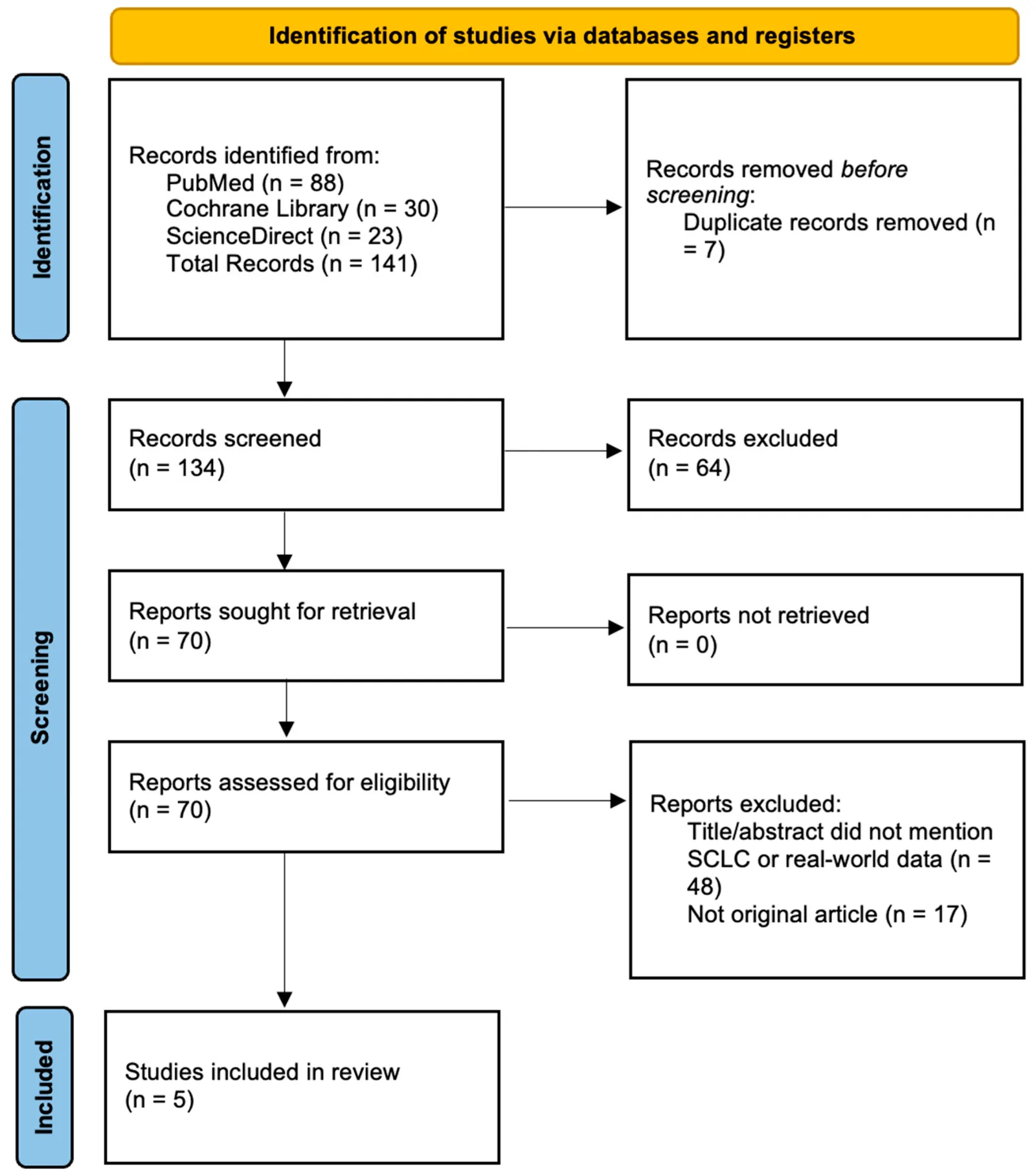
PRISMA flow diagram.

The eligibility criteria are summarized in Table 1. The study identification, screening, eligibility assessment, and inclusion processes are presented in the PRISMA flow diagram (Figure 1). The database-specific search strategies are summarized in Table 2.

### 2.3. Eligibility Criteria

Eligibility criteria were defined prior to screening and applied consistently during both the title/abstract and full-text review stages. We included original, non-randomized real-world studies enrolling adults (≥18 years) with SCLC who received tarlatamab in routine clinical practice, regardless of dosing schedule or step-up dosing approach. Studies were eligible if they reported at least one real-world effectiveness outcome (e.g., overall survival, progression-free survival/time-to-progression, response or best response, or time on treatment) and/or real-world safety outcomes with clear denominators, with particular emphasis on CRS and ICANS. Studies enrolling mixed populations (e.g., SCLC with other high-grade neuroendocrine carcinomas) were included only when SCLC-specific outcomes were extractable. We excluded interventional clinical trials (including post hoc trial subgroup analyses), reviews and editorials, regulatory approval summaries, translational/drug-resistance biology studies without real-world clinical outcomes, biomarker-only studies, clinical pharmacokinetic studies and exposure-response/exposure-safety modeling analyses, and indirect comparisons using external control arms. All sections of the eligibility criteria were recorded and applied prior to screening and can be found in Table 1 below.

### 2.4. Search Strategy

We systematically searched three electronic databases (PubMed, the Cochrane Library, and ScienceDirect) to identify real-world studies evaluating tarlatamab in SCLC. Searches were performed using keyword combinations centered on the terms “tarlatamab” and “small cell lung cancer,” with “real world evidence” added where applicable to enrich for observational outcomes studies. In PubMed, we first queried “Tarlatamab in Small Cell Lung Cancer” AND “Real World Evidence” (3 records) and then used a broader query “Tarlatamab” AND “Small Cell Lung Cancer” (85 records). In the Cochrane Library, we searched “Tarlatamab” AND “Small Cell Lung Cancer” (30 records). In ScienceDirect, we searched “Tarlatamab in Small Cell Lung Cancer” AND “Real World Evidence” (23 records) as provided in Table 2. Across databases, this yielded a total of 141 records prior to deduplication. Conference abstracts were eligible for inclusion when sufficient clinical outcome data were available for extraction. However, conference proceedings and abstract repositories were not systematically searched as separate information sources. Therefore, relevant conference abstracts or unpublished studies not indexed in the databases searched may have been missed. The search strategy was designed to identify published real-world clinical evidence regarding tarlatamab in SCLC using three electronic databases: PubMed, the Cochrane Library, and ScienceDirect. Both focused and broader keyword combinations were employed to identify relevant observational studies, with broader searches intended to capture publications that did not explicitly use the term “real-world evidence.” Although additional bibliographic databases and conference proceedings may contain relevant studies, these sources were not systematically searched. This represents a limitation of the review and may have resulted in the omission of emerging or unpublished real-world evidence.

The search strategy was designed to identify published clinical evidence regarding the real-world use of tarlatamab in SCLC. Both focused searches incorporating the term “real world evidence” and broader searches using “tarlatamab” and “small cell lung cancer” were performed to identify potentially eligible observational studies regardless of whether they were explicitly labeled as real-world evidence. Search expressions differed across databases according to their respective search interfaces. Conference abstracts were eligible for inclusion if they provided sufficiently detailed and extractable clinical outcomes; however, conference proceedings and abstract repositories were not systematically searched as independent information sources. Therefore, potentially relevant unpublished findings and conference abstracts not indexed in the selected databases may have been missed.

### 2.5. Study Selection Process

All records retrieved from the database searches were imported into EndNote for reference management and deduplication. Following duplicate removal, two reviewers independently screened titles and abstracts against the predefined eligibility criteria. Potentially eligible records were subsequently assessed through full-text review. Disagreements at either stage were resolved through discussion and consensus. Of the 141 identified records, 7 duplicates were removed, leaving 134 records for title and abstract screening. Sixty-four records were excluded at this stage, and seventy reports underwent full-text assessment. Following the exclusion of 65 reports that did not meet the eligibility criteria, 5 studies were included in the final qualitative synthesis. The study-selection process and reasons for exclusion are summarized in Figure 1.

### 2.6. Data Extraction

Data were extracted using a standardized data-collection form developed for this review. Two reviewers independently extracted study information and outcomes from each included paper, and any discrepancies were resolved through discussion and consensus. Extracted variables included study characteristics (publication year, country/center setting, study design, study period, and sample size), patient baseline characteristics (including disease setting when reported, prior therapies, performance status, and central nervous system involvement), and treatment details (tarlatamab dosing and step-up approach when described, and relevant supportive-care practices). Effectiveness outcomes extracted included response metrics as reported (e.g., ORR/BOR/DCR or best response categories), time-to-event outcomes such as overall survival and progression-free survival/time-to-progression when available, and time on treatment when reported. Safety data extraction focused on immune-mediated toxicities, particularly CRS and ICANS, including incidence and severity (any grade and grade ≥ 3 when provided), as well as other grade ≥3 adverse events, treatment discontinuation due to toxicity, and toxicity-management signals (e.g., tocilizumab or corticosteroid use) when reported. For studies enrolling mixed histologic populations, particular attention was given to the denominators used for effectiveness and safety outcomes. SCLC-specific outcomes were extracted wherever available, while results derived from combined histologic cohorts were explicitly identified as mixed-population estimates. Such findings were interpreted cautiously and were not considered definitive estimates of effectiveness or safety exclusively among patients with SCLC.

### 2.7. Outcomes

The outcomes of interest were selected to capture both real-world effectiveness and real-world safety of tarlatamab in SCLC. Effectiveness outcomes included overall survival (OS) and, when reported, progression-free survival or time-to-progression (PFS/TTP), with time zero defined as initiation of tarlatamab therapy as specified in each study. We also extracted tumor response outcomes as reported by the original authors, including objective response rate (ORR) where available, best overall response (BOR), disease control rate (DCR), and best response categories (complete response, partial response, stable disease, progressive disease). When response assessment methods were described, we recorded whether responses were determined by RECIST-based radiographic assessment or clinical/radiology documentation in routine practice. Safety outcomes focused on CRS and ICANS, including incidence and severity (any grade and grade ≥ 3 when available), timing when reported, and treatment-related discontinuation. Additional safety outcomes included grade ≥3 adverse events and serious adverse events as reported. Where available, we also extracted implementation-related measures relevant to routine care, such as use of tocilizumab and/or corticosteroids, need for hospitalization or intensive care, and other resource-use indicators described by the included studies.

### 2.8. Risk of Bias Assessment Methodology

The methodological quality of the included studies was evaluated using the Joanna Briggs Institute (JBI) Critical Appraisal Checklist for Case Series. This tool was selected to assess common methodological characteristics of the small, predominantly descriptive, non-randomized real-world reports included in the review. Two reviewers independently evaluated each study, considering the clarity of inclusion criteria, participant selection, reliability of condition identification, reporting of demographic and clinical characteristics, outcome ascertainment, and appropriateness of statistical analysis. Disagreements were resolved through discussion and consensus. The findings are summarized in Section 3.3. Given that some included studies were retrospective cohorts, including analyses with internal subgroup comparisons, the application of a single case-series appraisal tool may not fully capture confounding and other methodological limitations relevant to analytical observational designs.

### 2.9. Data Synthesis and Statistical Analysis

Given the small number of included studies and anticipated heterogeneity in patient populations (including special subgroups such as EGFR-mutant transformed SCLC), treatment delivery practices, and outcome definitions (particularly for response assessment and real-world progression), we planned a structured qualitative synthesis as the primary analytic approach. Study characteristics and key effectiveness and safety outcomes were summarized in evidence tables, and results were described narratively with attention to differences in study design, eligibility, baseline risk features (e.g., CNS involvement, performance status), and reporting methods.

Time-to-event outcomes (overall survival and progression-free survival/time-to-progression) were summarized using study-reported medians and associated confidence intervals when available. Response outcomes were summarized using study-reported proportions (e.g., ORR/BOR/DCR or best-response categories) with denominators as reported. Safety outcomes, including CRS and ICANS (any grade and grade ≥3 where available), were summarized as incidence proportions. When studies reported subgroup findings (e.g., CNS involvement, prophylactic tocilizumab use, or transformed SCLC), these results were extracted and presented separately to avoid inappropriate aggregation across clinically distinct populations.

Formal meta-analysis was considered only if at least two studies reported the same endpoint using sufficiently comparable definitions and populations. If pooling were deemed appropriate, we planned a random-effects model for proportions (e.g., CRS, ICANS, response) and would quantify statistical heterogeneity using I^2^. Because definitions of progression, response ascertainment, and follow-up varied across real-world studies, and because several outcomes were reported in evaluable subsets rather than the full treated cohort, we anticipated that quantitative pooling would often be inappropriate; in such cases, results were synthesized descriptively using ranges and study-level point estimates.

### 2.10. Assessment of Reporting Bias

Assessment of reporting bias (including publication bias) was planned; however, given the small number of included studies (n = 5) and the heterogeneity of study designs and outcome definitions, formal quantitative methods (e.g., funnel plots or Egger’s test) were not appropriate, as these approaches are generally unreliable when fewer than 10 studies contribute to an analysis. Instead, we evaluated reporting bias qualitatively by examining whether included studies provided complete reporting of prespecified outcomes (effectiveness and CRS/ICANS safety endpoints), whether outcomes were reported for the full treated cohort versus evaluable subsets, and whether key denominators and follow-up durations were clearly stated. We also attempted to mitigate selective reporting by using broad search terms and including multiple databases and sources (PubMed, Cochrane Library, and ScienceDirect), and by tracking conference-format reports when sufficient outcome data were available.

### 2.11. Certainty of Evidence

The certainty (overall quality) of evidence for key outcomes was evaluated using the Grading of Recommendations Assessment, Development and Evaluation (GRADE) approach. Certainty ratings were performed for the most clinically relevant endpoints, including overall survival, progression-free survival/time-to-progression, response outcomes, and the incidence of CRS and ICANS (any grade and grade ≥3 when available). Because the included evidence consisted of non-randomized real-world observational studies, the starting certainty was considered low, with potential downgrading based on risk of bias (e.g., selection bias, confounding, and outcome ascertainment), inconsistency across studies, indirectness (e.g., mixed histology cohorts or special subpopulations such as transformed SCLC), imprecision due to small sample sizes and limited follow-up, and the potential for publication/reporting bias. Where applicable, certainty could be upgraded for large magnitude of effect or consistent dose–response patterns, although such upgrading was not expected to be common in this evidence base. Final GRADE judgments were summarized qualitatively to contextualize interpretation of study-level findings.

## 3. Results

### 3.1. Study Selection

The database search identified 141 records across PubMed, the Cochrane Library, and ScienceDirect. After removal of 7 duplicates, 134 unique records underwent title and abstract screening, of which 64 were excluded. The remaining 70 articles were assessed in full text for eligibility; 65 were excluded because they did not meet inclusion criteria, most commonly because they did not report real-world outcomes or were clinical trials, reviews/editorials, translational or drug-resistance biology studies, pharmacokinetic or exposure-response analyses, external control/indirect comparison studies, post hoc subgroup analyses, biomarker-only studies, or regulatory approval summaries. Ultimately, five studies met the eligibility criteria and were included in the qualitative synthesis, as shown in Figure 1.

### 3.2. Study Characteristics

All five included studies were non-randomized, real-world reports describing outcomes of patients treated with tarlatamab in routine practice, and all were retrospective in design, encompassing single-center and multi-center cohorts. The University of Virginia study by Bolte et al. was a retrospective observational cohort study evaluating all patients treated with tarlatamab between May and October 2024, including 21 SCLC patients and one DLL3-positive atypical carcinoid (included in the overall safety cohort), and it specifically captured patients with untreated brain metastases and other features that would have excluded them from prior registrational trials [5]. A multi-institution retrospective cohort study by Fenton et al. (University of Maryland Medical Center and University Hospitals/Case Western Reserve) evaluated 48 patients treated with tarlatamab as standard of care between July 2024 and May 2025, including patients with SCLC and LCNEC, and defined a prespecified efficacy analysis subset (n = 30) based on availability of post-treatment imaging [6]. Cooper et al. reported a six-institution retrospective chart review focused on a biologically distinct subgroup, EGFR-mutant transformed SCLC, including 16 patients, of whom 75% had Eastern Cooperative Oncology Group (ECOG) 0–1 and 50% had brain metastases at tarlatamab initiation [7]. The University of Kansas Cancer Center cohort study by Mullangi et al. examined tarlatamab use in higher-risk real-world populations treated between May and December 2024, including 21 SCLC patients (and a small extrapulmonary cohort), with a high prevalence of CNS involvement (66.6%), baseline oxygen requirement (23.8%), and ECOG ≥ 2 (23.8%) [8]. Finally, Joshi et al. reported a single-center retrospective study from Moffitt Cancer Center (May 2024–February 2025; n = 40) focused on operational delivery and early toxicity, including an internal comparison of patients who received prophylactic tocilizumab (n = 10) versus those who did not (n = 30) during step-up dosing, alongside modeling of clinical predictors of CRS/ICANS in the non-prophylaxis group [9]. The characteristics of the five included real-world studies are summarized in Table 3.

### 3.3. Risk of Bias Assessment

Risk of bias was assessed using the Joanna Briggs Institute (JBI) critical appraisal tools, with checklist selection guided by the methodological design of each included study. Retrospective observational cohorts were evaluated using the JBI Critical Appraisal Checklist for Cohort Studies, while studies meeting the methodological characteristics of case series were evaluated using the JBI Critical Appraisal Checklist for Case Series. Two reviewers independently assessed each study, and disagreements were resolved through discussion and consensus. The assessment considered relevant methodological domains, including participant selection, outcome measurement, completeness of follow-up, statistical analysis, and, where applicable, confounding. The findings were summarized according to the applicable checklist and used to inform the interpretation of the evidence, as presented in Table 4.

### 3.4. Effectiveness Outcomes

Across the included real-world studies, effectiveness was reported using response-based endpoints (objective response, best overall response, and disease control) and time-to-event outcomes (PFS and OS). However, response ascertainment and analytic denominators differed across studies, with several analyses restricted to response-evaluable subsets rather than the entire treated population. To facilitate interpretation, the total treated population and response-evaluable population are reported separately for each study where available. Such restrictions may introduce selection or survivorship bias, particularly when patients without post-treatment imaging or those who experienced early clinical deterioration were excluded from response analyses.

In the University of Virginia cohort reported by Bolte et al., 22 patients received tarlatamab, including 21 patients with SCLC and one patient with atypical carcinoid. Antitumor activity was assessed in the 21 patients with SCLC, excluding the atypical carcinoid patient included in the overall safety cohort. Using investigator-assessed RECIST 1.1, best overall response among the 21 SCLC patients included partial response (PR) in 42.9% (9/21), stable disease (SD) in 28.6% (6/21), and progressive disease (PD) in 19.0% (4/21), with two patients (9.5%; 2/21) dying before the first postbaseline scan. The objective response rate (ORR) was 42.9% (9/21), and the disease control rate (DCR) was 71.4% (15/21). Median PFS was 2.7 months, while median OS was not reached at the time of analysis [5].

In the University of Kansas Cancer Center cohort reported by Mullangi et al., 21 patients with SCLC received tarlatamab, of whom 17 (81.0%; 17/21) were evaluable for response. Among these 17 response-evaluable patients, best response included PR in 35.2% (6/17), SD in 17.6% (3/17), and PD in 47.0% (8/17). Thus, the response percentages reflect the 17 evaluable patients rather than the entire treated SCLC cohort of 21 patients. Median PFS was 2.2 months and median OS was 4.1 months, although the authors noted that efficacy estimates were immature because of short follow-up [8].

In the two-center real-world cohort reported by Fenton et al., 48 patients received at least one dose of tarlatamab. However, the reported effectiveness analysis was restricted to 30 patients (62.5%; 30/48) with available post-treatment imaging. Among these 30 evaluable patients, objective response was observed in 66.7% (20/30), including complete response in 6.7% (2/30), while the disease control rate was approximately 73%. When considering the entire treated cohort of 48 patients, the 20 documented responses correspond to 41.7% (20/48) of treated patients. This figure represents the minimum documented response proportion in the full cohort and should not be interpreted as a formal intention-to-treat objective response rate, as response outcomes were unavailable for 18 patients. Median PFS was 4.9 months (95% CI 4–NR) and median OS was not reached (95% CI 4.8–NR) at a median follow-up of 5.7 months. Median duration of response among responders was 3.8 months (95% CI 1.8–5.8). Restriction of the effectiveness analysis to patients with post-treatment imaging may introduce selection or survivorship bias, particularly if patients experiencing early clinical deterioration or death were less likely to undergo subsequent imaging [6].

In the biologically distinct population of EGFR-mutant transformed SCLC reported by Cooper et al., 16 patients received tarlatamab, of whom 14 (87.5%; 14/16) were response-evaluable. Among these 14 patients, clinically assessed best response included partial response (PR) in 7.1% (1/14), mixed response in 21.4% (3/14), stable disease (SD) in 7.1% (1/14), and progressive disease (PD) in 64.3% (9/14). These response estimates were calculated using the 14 evaluable patients rather than the full treated cohort of 16 patients. Median OS from tarlatamab initiation was 2.7 months (range 0.75–8), with a median follow-up of 4 months [7].

In the Moffitt Cancer Center cohort reported by Joshi et al., 40 patients received tarlatamab, including 30 patients who did not receive prophylactic tocilizumab and 10 patients who received prophylaxis. At the April 2025 data cutoff, response outcomes were available for 23 of 30 patients (76.7%) in the non-prophylaxis group and 8 of 10 patients (80.0%) in the prophylaxis group. Among the 23 response-evaluable patients without prophylaxis, 47.8% (11/23) achieved a physician-assessed disease response and 52.1% (12/23) experienced progression. Among the eight response-evaluable patients receiving prophylaxis, 50.0% (4/8) achieved disease response and 50.0% (4/8) experienced progression. Median PFS was reported as 1.8 months (95% CI 1.1–3.7) without prophylaxis and 1.7 months (95% CI 1.1–2.5) with prophylaxis after a median follow-up of 7.5 months. These response proportions should be interpreted in the context of the evaluable subsets and the non-randomized nature of the subgroup comparison [9]. To facilitate comparison across studies, Table 5 summarizes the treated populations, response-evaluable populations, effectiveness outcomes, and principal safety findings. Response assessment was restricted to evaluable subsets in several studies, and the number of patients without evaluable response data varied between cohorts. Consequently, reported response rates should be interpreted according to their respective denominators and should not be directly compared across heterogeneous study populations.

### 3.5. Comparator Information

None of the included studies incorporated an external comparator arm, such as tarlatamab versus another systemic therapy, and effectiveness and safety outcomes were therefore interpreted within single-arm real-world cohorts. Bolte et al., Mullangi et al., Cooper et al., and Fenton et al. each reported descriptive outcomes among patients treated with tarlatamab in routine clinical practice without a concurrent control group. Joshi et al. likewise did not include an external comparator, although the study reported a non-randomized internal subgroup comparison between patients who received prophylactic tocilizumab and those who did not. Because this subgroup analysis was observational and susceptible to confounding by indication, it was considered exploratory and hypothesis-generating rather than a causal estimate of the effect of prophylactic tocilizumab.

### 3.6. Synthesis Statement

In this systematic review of real-world tarlatamab use in SCLC, five observational studies met the eligibility criteria, comprising predominantly retrospective single-center or limited multicenter cohorts with heterogeneous populations and outcome ascertainment. Among cohorts evaluating predominantly de novo SCLC, clinically meaningful antitumor activity was observed, although efficacy estimates were frequently restricted to response-evaluable subsets and limited by short follow-up. In contrast, Cooper et al. evaluated EGFR-mutant transformed SCLC, a biologically distinct population in which substantially less favorable outcomes were reported, including a partial response in only 1 of 14 response-evaluable patients and median overall survival of 2.7 months [7]. These findings were therefore interpreted separately and were not incorporated into generalized conclusions regarding effectiveness in conventional de novo ES-SCLC. Safety findings demonstrated that CRS and ICANS remained clinically important toxicities in routine practice, with observed incidence varying across heterogeneous cohorts. None of the included studies incorporated an external comparator arm, and the only internal, non-randomized subgroup comparison involving prophylactic tocilizumab was considered exploratory. Overall, the available evidence supports early signals of antitumor activity in conventional de novo SCLC while highlighting uncertainty regarding effectiveness in EGFR-mutant transformed disease and the need for larger prospective studies with standardized outcome definitions.

In addition to overall CRS and ICANS incidence, the included studies provided preliminary information regarding the timing of immune-mediated toxicities and their management during tarlatamab administration. Mullangi et al. reported a more favorable safety profile following subsequent doses compared with initial administration, suggesting that the risk of CRS and ICANS may vary during step-up dosing [8]. However, these observations were derived from a small retrospective cohort and were insufficient to establish the safety of abbreviated monitoring or outpatient administration.

Joshi et al. evaluated the use of prophylactic tocilizumab during step-up dosing in 40 patients, including 30 who did not receive prophylaxis and 10 who did. Following cycle 1, day 1 administration, CRS occurred in 53.3% (16/30) of patients without prophylaxis compared with 10.0% (1/10) of patients receiving prophylaxis, while ICANS occurred in 23.3% (7/30) and 0% (0/10), respectively. In exploratory predictive analyses of the 30 patients who did not receive prophylaxis, elevated lactate dehydrogenase (LDH) and liver metastases were independently associated with CRS, while LDH was also associated with grade ≥2 CRS. Diabetes mellitus and cardiovascular disease were independently associated with ICANS [9]. Given the small sample size, observational design, and potential for confounding, these findings were considered hypothesis-generating rather than definitive evidence supporting prophylactic tocilizumab or individual risk prediction.

### 3.7. Certainty of Evidence (GRADE Assessment)

The certainty of evidence for the principal effectiveness and safety outcomes was evaluated using the Grading of Recommendations Assessment, Development and Evaluation (GRADE) framework. Given that all five included studies were retrospective, non-randomized observational reports without randomized treatment comparisons, the initial certainty was considered low. The evidence was further evaluated for risk of bias, inconsistency, indirectness, imprecision, and publication bias.

Overall, the certainty of evidence was judged to be very low across the principal effectiveness outcomes, including objective response, progression-free survival (PFS), and overall survival (OS). These judgments primarily reflected the small sample sizes, heterogeneous patient populations, variable follow-up durations, and differences in outcome assessment. Several studies restricted effectiveness analyses to response-evaluable subsets, introducing potential selection bias and limiting confidence in the reported estimates. Furthermore, the absence of concurrent treatment comparators prevented reliable conclusions regarding comparative effectiveness.

The certainty of evidence for cytokine release syndrome (CRS) and immune effector cell-associated neurotoxicity syndrome (ICANS) was also judged to be very low. Although these adverse events were consistently reported, differences in baseline patient characteristics, toxicity grading, prophylactic interventions, and institutional monitoring practices complicated comparisons across studies. Small sample sizes and limited reporting of severe adverse events further reduced precision. The available evidence therefore provides descriptive estimates of real-world toxicity rather than definitive estimates of comparative safety.

The outcome-level certainty assessments and principal considerations informing these judgments are summarized in Table 6. These ratings indicate that the reported estimates may change substantially as larger, prospective, and methodologically standardized real-world studies become available.

## 4. Discussion

This systematic review synthesized the available real-world evidence regarding the effectiveness and safety of tarlatamab in patients with small cell lung cancer (SCLC). Five retrospective observational studies met the eligibility criteria, providing early insights into treatment outcomes in populations often underrepresented in prospective clinical trials. Overall, the findings suggest that tarlatamab demonstrates clinically meaningful antitumor activity in previously treated de novo SCLC, although effectiveness estimates vary across studies and remain limited by small sample sizes, heterogeneous patient populations, and differences in outcome ascertainment. Cytokine release syndrome (CRS) and immune effector cell-associated neurotoxicity syndrome (ICANS) were frequently reported, highlighting the importance of careful patient selection, early monitoring, and appropriate toxicity management. Importantly, outcomes in EGFR-mutant transformed SCLC were substantially less favorable and should be interpreted separately from those in conventional de novo SCLC.

### 4.1. Real-World Effectiveness

The real-world studies included in this review provide preliminary evidence that the antitumor activity of tarlatamab extends beyond the selected populations enrolled in the DeLLphi clinical trials. In DeLLphi-301, tarlatamab demonstrated an objective response rate (ORR) of 40% and median progression-free survival (PFS) of 4.9 months in the 10 mg cohort, while DeLLphi-304 subsequently demonstrated improved overall survival (OS) compared with chemotherapy in previously treated SCLC [3,4]. Although the observational studies were not designed to establish comparative effectiveness, several reported meaningful response and disease-control outcomes in routine clinical practice. These findings should also be considered within the broader context of evolving systemic treatment strategies and therapeutic sequencing in SCLC [10].

Among patients with predominantly de novo SCLC, Bolte et al. reported an ORR of 42.9% (9/21) and disease control rate (DCR) of 71.4% (15/21), despite including patients with adverse baseline characteristics and frequent ineligibility for registrational trials [5]. Similarly, Fenton et al. reported an ORR of 66.7% (20/30) and DCR of approximately 73% among 30 response-evaluable patients from a total treated cohort of 48 patients [6]. Mullangi et al. observed partial responses in 35.2% (6/17) of response-evaluable SCLC patients from a treated cohort of 21, with median PFS and OS of 2.2 and 4.1 months, respectively [8]. These findings suggest that clinical activity can be observed in broader populations; however, comparisons across studies are limited by differences in eligibility, follow-up, and the use of response-evaluable rather than treated-population denominators. In particular, excluding patients without post-treatment imaging may introduce selection or survivorship bias and potentially overestimate treatment activity.

An important distinction emerged in EGFR-mutant transformed SCLC. Cooper et al. reported a partial response in only 1 of 14 response-evaluable patients from a treated cohort of 16, with median OS of 2.7 months [7]. These findings may reflect biological differences between de novo and transformed SCLC. Although TP53 and RB1 inactivation are characteristic molecular alterations in conventional SCLC, these alterations are also frequently observed in EGFR-mutant transformed SCLC and therefore do not distinguish the two entities. Transformed SCLC arises through lineage plasticity and clonal evolution of a preexisting EGFR-mutant lung adenocarcinoma, frequently retaining the original EGFR driver alteration while acquiring a neuroendocrine phenotype [11,12]. Differences in tumor evolution, molecular heterogeneity, and potentially DLL3 expression may contribute to variability in treatment response [13]. However, the relationship between these molecular characteristics and tarlatamab effectiveness remains incompletely understood. Accordingly, the less favorable outcomes observed in EGFR-mutant transformed SCLC should be considered hypothesis-generating and should not be generalized to conventional de novo SCLC.

Molecular heterogeneity may also influence responses to DLL3-directed therapies. SCLC exhibits distinct transcriptional programs involving ASCL1, NEUROD1, and POU2F3, with variable neuroendocrine differentiation and DLL3 expression [14,15,16]. However, none of the five included real-world studies systematically evaluated transcriptional subtypes or their association with tarlatamab outcomes. Therefore, the potential predictive significance of these molecular features remains speculative and warrants investigation in future studies.

Central nervous system (CNS) involvement represents an important clinical consideration in lung cancer, with brain metastases contributing substantially to disease burden and clinical outcomes in both small cell and selected molecular subtypes of non-small cell lung cancer [17,18]. The inclusion of patients with CNS involvement therefore represents an important contribution of the real-world tarlatamab literature. Bolte et al. and Mullangi et al. included patients with untreated or recurrent brain metastases and described encouraging clinical outcomes in selected cases [5,8]. However, standardized intracranial response assessments were limited, and some patients received concurrent or prior radiation therapy. Therefore, the available evidence supports the feasibility of tarlatamab treatment in selected patients with CNS disease but does not establish definitive intracranial efficacy. The broader clinical importance of CNS involvement is further supported by population-based evidence describing the burden and management of brain metastases in SCLC [19]. Additionally, molecular investigations of EGFR-mutant transformed SCLC have highlighted the relevance of DLL3 expression and treatment heterogeneity in this distinct population [20].

### 4.2. Safety and Toxicity Management

CRS and ICANS were the principal immune-mediated toxicities identified across the included studies. Bolte et al. reported CRS and ICANS in 72.7% and 40.9% of treated patients, respectively, while Mullangi et al. reported corresponding rates of 61.9% and 47.6% in their SCLC cohort [5,8]. Fenton et al. similarly observed CRS in 54.2% and ICANS in 33.3% of patients [6]. Although some observational cohorts reported numerically higher toxicity rates than those observed in DeLLphi-301, these differences should not be interpreted as evidence of a definitively higher underlying toxicity risk in routine practice. Variations in patient characteristics, baseline CNS involvement, toxicity ascertainment, grading practices, follow-up duration, and institutional monitoring protocols may contribute to the observed differences [3,5,6,8]. Mechanistically, CRS associated with bispecific T-cell engagers results from treatment-induced T-cell activation and subsequent release of inflammatory mediators, including interleukin-6 (IL-6), interferon-γ, and other cytokines. A particularly important finding of this review was the numerically higher incidence of ICANS reported in several real-world cohorts compared with prospective clinical trial data. In the DeLLphi-301 trial, ICANS was reported in 5.3% of patients in the 10 mg safety population [21], whereas Bolte et al., Mullangi et al., and Fenton et al. reported incidences of 40.9%, 47.6%, and 33.3%, respectively [5,6,8]. Joshi et al. additionally reported ICANS in 23.3% of patients who did not receive prophylactic tocilizumab following cycle 1, day 1 dosing [9]. Although these findings suggest that neurologic toxicity may be a greater challenge in routine clinical practice, direct comparisons should be interpreted cautiously. Real-world cohorts included patients with a substantial CNS disease burden, impaired performance status, and other characteristics underrepresented in prospective trials. Differences in neurologic assessment, toxicity grading, monitoring intensity, and follow-up may also contribute to the observed variation. Importantly, these observational findings do not establish a higher underlying risk of ICANS outside clinical trials. Nevertheless, the consistently elevated rates reported in several real-world cohorts emphasize the importance of careful neurologic monitoring during tarlatamab administration and support further investigation into clinical predictors of ICANS in broader patient populations.

Baseline CNS disease was particularly relevant to the interpretation of neurologic toxicity. Bolte et al. and Fenton et al. described ICANS among patients with brain metastases, suggesting that preexisting neurologic disease may complicate toxicity assessment and management [5,6]. However, these observational findings do not establish a causal relationship between brain metastases and ICANS. In addition, baseline oxygen requirements, opioid exposure, and other comorbidities may influence the assessment or grading of CRS and neurologic adverse events [5,8].

The timing and potential prevention of immune-mediated toxicities represent important considerations for optimizing tarlatamab administration. Mullangi et al. reported a more favorable safety profile following subsequent doses, suggesting that monitoring requirements might eventually be individualized according to patient characteristics and treatment tolerance [8]. However, these preliminary findings do not independently justify modifying established monitoring protocols. Joshi et al. reported lower rates of CRS and ICANS among patients receiving prophylactic tocilizumab, although the non-randomized comparison was limited by the small prophylaxis group and potential confounding [9]. Their exploratory analyses also identified elevated LDH and liver metastases as predictors of CRS, while diabetes mellitus and cardiovascular disease were associated with ICANS. These associations require external validation before incorporation into clinical risk-stratification models. Collectively, these findings suggest potential opportunities for individualized toxicity management but do not establish the safety or effectiveness of routine prophylactic tocilizumab or abbreviated monitoring. Prospective studies are needed to determine whether such strategies can safely reduce treatment-related toxicity and healthcare resource utilization.

### 4.3. Strengths and Limitations

A principal strength of this review is its focus on real-world tarlatamab use, including patients with characteristics frequently underrepresented in clinical trials, such as reduced performance status, CNS involvement, and substantial comorbidity burden. By synthesizing findings from five observational studies, this review provides a structured assessment of early effectiveness, toxicity, and implementation challenges encountered in routine practice.

Nevertheless, several limitations warrant consideration. All included studies were retrospective, non-randomized, and relatively small, with no external comparator arms. Differences in patient populations, treatment histories, response definitions, follow-up durations, and analytic denominators limited direct comparisons and precluded meaningful quantitative pooling. Some studies restricted efficacy analyses to patients with available post-treatment imaging, introducing potential selection and survivorship bias [5,6,7,8,9]. Furthermore, retrospective toxicity ascertainment and variable institutional monitoring practices complicated comparisons of CRS and ICANS incidence across cohorts and with prospective clinical trials. An additional consideration is the possibility of overlapping patient populations across institutional and multicenter reports conducted during similar periods. Although the included studies described separate cohorts, patient-level data were not available to definitively exclude potential duplication. Consequently, the combined sample sizes should not necessarily be interpreted as representing entirely unique patients. However, because no quantitative pooling was performed, potential overlap did not directly affect a pooled effect estimate.

The certainty of evidence was therefore very low for the principal effectiveness and safety outcomes, as summarized in the GRADE assessment [22] and summarized in Table 6. These limitations indicate that the findings should be interpreted as descriptive, hypothesis-generating evidence rather than definitive estimates of comparative treatment effectiveness or safety. The small and rapidly evolving evidence base also introduces potential publication and reporting bias, particularly given the concentration of early studies in specialized academic centers [22,23]. Additionally, the small and rapidly evolving evidence base introduces potential publication and reporting bias, as early real-world findings may be disproportionately represented by studies from specialized academic centers, while smaller or less favorable experiences may remain unpublished.

Additional limitations relate to the literature search and methodological assessment. The search was restricted to three electronic databases and did not systematically include additional bibliographic databases or conference proceedings, potentially limiting the comprehensiveness of the identified evidence. Furthermore, the small and rapidly evolving real-world literature raises the possibility of relevant studies emerging after the final search date. The inclusion of mixed histologic cohorts and the lack of patient-level information to definitively exclude overlap between institutional reports also warrant consideration. Finally, although the JBI Case Series Checklist provided a consistent framework for assessing the included studies, its application to retrospective cohort designs may not fully account for confounding and other biases associated with observational comparisons. These limitations reinforce the need for cautious interpretation of the findings. Future observational studies should prioritize standardized reporting of patient characteristics, outcome definitions, and methodological limitations in accordance with established reporting recommendations, including the STROBE guidelines [24,25].

### 4.4. Clinical Implications and Future Directions

Tarlatamab represents an established treatment option for previously treated extensive-stage small cell lung cancer (ES-SCLC), supported by prospective evidence demonstrating meaningful antitumor activity in DeLLphi-301 and improved overall survival compared with chemotherapy in DeLLphi-304 [3,4]. Its incorporation into clinical practice has been supported by regulatory milestones, including accelerated and subsequent traditional approval by the U.S. Food and Drug Administration, alongside evolving recommendations for systemic treatment of SCLC [26,27,28]. The real-world studies included in this review provide preliminary evidence that treatment activity may extend to broader patient populations, including individuals with adverse baseline characteristics. However, these observational findings should not be interpreted as establishing treatment superiority, optimal sequencing, or equivalent effectiveness across biologically distinct SCLC subgroups. In particular, the limited outcomes reported in EGFR-mutant transformed SCLC warrant separate consideration [7].

The findings also highlight practical considerations regarding the management of immune-mediated toxicities. Structured monitoring, early recognition, and prompt management of cytokine release syndrome (CRS) and immune effector cell-associated neurotoxicity syndrome (ICANS) remain important components of tarlatamab administration [21]. Although retrospective studies suggest that toxicity may be concentrated during initial step-up dosing, these observations do not independently establish the safety of abbreviated monitoring or outpatient administration beyond existing prescribing recommendations and institutional protocols [5,8]. Decisions regarding treatment setting and monitoring intensity should therefore continue to follow established safety guidance rather than rely solely on preliminary real-world observations.

Similarly, prophylactic tocilizumab and individualized risk-adapted toxicity-management approaches remain investigational. Joshi et al. reported encouraging findings among patients receiving prophylactic tocilizumab; however, the small sample size, non-randomized design, and potential for confounding limit causal interpretation [9]. Proposed clinical predictors of CRS and ICANS require external validation before they can support standardized risk-stratification strategies. Consequently, routine prophylactic tocilizumab or modified monitoring protocols cannot be recommended solely on the basis of these early observational findings.

Future research should prioritize prospective multicenter studies evaluating real-world effectiveness, standardized toxicity assessment, and alternative treatment-delivery strategies. Particular attention should be directed toward patients with CNS involvement, impaired performance status, and EGFR-mutant transformed SCLC. An important priority is the integration of molecular profiling into prospective real-world registries, including assessment of ASCL1, NEUROD1, POU2F3, and YAP1-associated transcriptional programs alongside DLL3 immunohistochemistry. Such data may help clarify whether tumor subtype, DLL3 expression, and lineage plasticity influence tarlatamab response, resistance, or survival. Prospective evaluation of outpatient administration, prophylactic interventions, and validated risk-prediction models will also be necessary before these approaches can be incorporated into broader clinical recommendations [7,8,9,14,15,16]. Beyond tarlatamab, emerging approaches targeting DLL3 and other tumor-associated antigens, including antibody–drug conjugates and bispecific T-cell engagers, represent additional areas of therapeutic development in SCLC [29,30,31].

## 5. Conclusions

In conclusion, the currently available real-world evidence suggests that tarlatamab demonstrates clinically meaningful antitumor activity in previously treated de novo extensive-stage small cell lung cancer, including patients who are frequently underrepresented or excluded from prospective clinical trials. However, these findings should not be generalized to EGFR-mutant transformed SCLC, a biologically distinct subgroup in which substantially less favorable outcomes have been reported. The available studies also highlight the importance of cytokine release syndrome and neurologic toxicity monitoring in routine practice, although differences in patient characteristics, toxicity ascertainment, and institutional monitoring limit direct comparisons with clinical trial safety outcomes. Given the retrospective design, small sample sizes, absence of external comparator arms, and heterogeneity across cohorts, these findings should be interpreted cautiously. Further prospective studies are needed to clarify the effectiveness of tarlatamab in distinct SCLC subpopulations, optimize toxicity mitigation, and better define its role in treatment sequencing.

## Figures and Tables

**Table 1:** PICO-TT framework.

Domain	Inclusion	Exclusion
Population	Adults (≥18 years) with SCLC treated in routine clinical practice. Mixed neuroendocrine cohorts (e.g., SCLC + LCNEC/high-grade NEC) eligible only if SCLC outcomes are extractable. Includes patients with baseline CNS metastasis and/or reduced performance status if reported.	Pediatric populations. Non-SCLC-only cohorts. Mixed histology cohorts where SCLC-specific outcomes cannot be extracted. Case reports/very small case series (n < 10).
Intervention	Tarlatamab (AMG 757; IMDELLTRA) administered in a real-world setting (standard of care or expanded access/compassionate use when reported as routine practice), any dose/schedule/step-up regimen.	Studies where tarlatamab was not administered. Preclinical/in vitro/in vivo studies without human treatment outcomes.
Comparator	Comparator not required (single-arm RWE eligible). If present, internal subgroup comparisons (e.g., prophylactic tocilizumab vs. none) or observational comparisons are eligible and described separately.	Indirect treatment comparisons/external control arm studies (e.g., MAIC/external controls) without real-world tarlatamab-treated cohorts.
Outcome(s)	At least one real-world effectiveness outcome (rwOS, rwPFS/TTP, response/BOR/DCR, time on treatment) and/or real-world safety outcomes with denominators, including CRS and/or ICANS (any grade and grade ≥3 when available).	Studies without extractable real-world clinical outcomes (e.g., narrative reviews, editorials, approval summaries). Safety signal studies without denominators (e.g., FAERS disproportionality) if no incidence can be estimated. Biomarker-only studies without clinical outcomes.
Timing	Any follow-up duration; outcomes measured from tarlatamab initiation when reported. No restriction on year; includes contemporary post-availability period.	
Type of Study	Real-world observational studies: retrospective/prospective cohorts, registries, EHR/claims studies, institutional series (n > 10), expanded access cohorts with outcomes. Full texts and conference abstracts eligible if sufficient data are extractable.	Interventional clinical trials (phase 1–3 RCTs and single-arm trials) and post hoc trial subgroup analysis. PK/PD studies, clinical pharmacokinetics, exposure-efficacy/exposure-safety modeling. Translational resistance biology without real-world outcomes.

**Table 2:** Summary of search strategies used across databases.

Database	Keywords	Search Strategy	Filters	Search Result
PubMed	“tarlatamab in small cell lung cancer AND real world evidence” “tarlatamab AND small cell lung cancer”	Advanced filtered search and MeSH	None	88
Cochrane Library	“tarlatamab AND small cell lung cancer”	Advanced filtered search	None	30
ScienceDirect	“tarlatamab in small cell lung cancer AND real world evidence”	Advanced filtered search	None	23

**Table 3:** Study characteristics of included real-world studies.

Study	Design/Setting	Study Period	Population	Sample Size	Key Baseline Features Reported
Bolte et al., 2025 [5]	Retrospective observational cohort; University of Virginia	May–October 2024	SCLC (plus DLL3 + atypical carcinoid included in safety only)	n = 22 treated (SCLC n = 21; carcinoid n = 1)	Median age 66; ES-SCLC 85.7%; brain mets 40.9%; liver mets 63.6% (14/22); 81.8% trial-ineligible; ECOG ≥ 2 18.2%; 72.7% developed CRS; 40.9% developed ICANS
Mullangi et al., 2025 [8]	Retrospective chart review; University of Kansas Cancer Center	May–December 2024	SCLC (cohort 1) ± EPSCC (cohort 2)	SCLC n = 21; EPSCC n = 3	In SCLC cohort: CNS involvement 66.6%; baseline oxygen 23.8%; ECOG ≥ 2 23.8%; 61.9% developed CRS; 47.6% developed ICANS
Fenton et al., 2026 [6]	Retrospective multi-institution cohort; University of Maryland Medical Center + Case Western University Hospital	July 2024–May 2025	Previously treated ES-SCLC and LCNEC (mixed cohort)	N = 48 treated; efficacy analysis n = 30 (post-treatment imaging)	Median age 68; ECOG ≥ 2 33%; intracranial involvement 67%; 54.2% developed CRS; 33.3% developed ICANS
Cooper et al., 2025 [7]	Retrospective chart review across 6 U.S. academic institutions	Multi-institution retrospective (timeframe not required for inclusion)	EGFR-mutant transformed SCLC	N = 16 (response evaluable n = 14)	Median age at SCLC transformation 56.5; ECOG 0–1 75%; brain mets 50% (most treated with RT); 56.3% (9/16) developed CRS; no patients experienced ICANS
Joshi et al., 2026 [9]	Retrospective single-center cohort; Moffitt Cancer Center	May 2024–February 2025	SCLC and high-grade neuroendocrine lung cancer	N = 40; no prophylaxis (n = 30); prophylactic tocilizumab (n = 10)	Median age 66.5 years; male 55%; ECOG 0–1 in 87.5%. Following C1D1, CRS occurred in 53.3% (16/30) without prophylaxis versus 10% (1/10) with prophylaxis; ICANS occurred in 23.3% (7/30) versus 0% (0/10), respectively.

For Joshi et al. [9], CRS and ICANS rates refer to events observed following cycle 1, day 1 (C1D1) dosing. Rates are presented separately for the non-prophylaxis and prophylactic tocilizumab groups. Comparisons between these groups are observational and should be interpreted cautiously.

**Table 4:** JBI case series checklist.

JBI Case Series Items	Bolte et al., 2025 [5]	Mullangi et al., 2025 [8]	Fenton et al., 2026 [6]	Cooper et al., 2025 [7]	Joshi et al., 2026 [9]
Clear inclusion criteria?	Y	Y	Y	Y	Y
Condition measured reliably?	Y	Y	Y	Y	Y
Valid condition identification?	Y	Y	Y	Y	Y
Consecutive inclusion?	Y	U	U	U	U
Complete inclusion?	U	U	U	U	U
Demographics clearly reported?	Y	Y	Y	Y	Y
Clinical information clearly reported?	Y	Y	Y	Y	Y
Outcomes/follow-up clearly reported?	Y	Y	Y	Y	Y
Site(s)/setting clearly described?	Y	Y	Y	Y	Y
Appropriate statistical analysis?	Y	Y	Y	Y	Y

Y = Yes; U = Unclear. The methodological appraisal was conducted using the Joanna Briggs Institute (JBI) Critical Appraisal Checklist for Case Series. Item-level judgments are presented individually, and methodological limitations were interpreted qualitatively rather than through a numerical summary score.

**Table 5:** Summary of real-world effectiveness and safety outcomes of tarlatamab.

Study	Treated (n)	Response-Evaluable (n)	Response Outcomes	PFS/OS (Months)	CRS/ICANS
Bolte et al. [5]	22 (21 SCLC)	21 SCLC *	ORR 42.9%; DCR 71.4%	2.7/NR	72.7%/40.9%
Mullangi et al. [8]	24 (21 SCLC)	17 SCLC	PR 35.3%; SD 17.6%	2.2/4.1	61.9%/47.6%
Fenton et al. [6]	48	30	ORR 66.7%; DCR 73%	4.9/NR	54.2%/33.3%
Cooper et al. [7]	16	14	PR 7.1%; SD 7.1%	NR/2.7	56.3%/0%
Joshi et al. [9]	40	23 without prophylaxis; 8 with prophylaxis	Response 47.8% vs. 50%	1.8 vs. 1.7/NR	CRS 53.3% vs. 10%; ICANS 23.3% vs. 0%

Abbreviations: SCLC, small cell lung cancer; ORR, objective response rate; DCR, disease control rate; PR, partial response; SD, stable disease; PFS, progression-free survival; OS, overall survival; CRS, cytokine release syndrome; ICANS, immune effector cell-associated neurotoxicity syndrome; NR, not reported or not reached, as applicable. For Joshi et al., toxicity rates refer to cycle 1, day 1, and are presented for the non-prophylaxis and prophylactic tocilizumab groups, respectively. Mixed-histology cohort estimates are not SCLC-specific. * Bolte et al. included two patients who died before the first scan in the 21-patient response denominator. Percentages use the original studies’ reported populations; mixed-histology and C1D1 safety estimates are not directly comparable.

**Table 6:** GRADE summary of findings.

Outcome	Studies	Summary of Reported Findings	Principal Reasons for Downgrading	GRADE Certainty
Objective response/best response	5	Response varied markedly; ORR or partial response estimates ranged from 7% to 66.7% in the studies reporting these measures	Risk of bias from evaluable-subset selection; imprecision; indirectness from transformed/mixed histologies and variable response assessment	⊕◯◯◯ Very low
Progression-free survival	4	Median PFS ranged from 1.7 to 4.9 months across reported analyses	Risk of bias, including selection and variable progression assessment; imprecision; heterogeneous follow-up	⊕◯◯◯ Very low
Overall survival	4	Median OS was 2.7–4.1 months in studies where reached; not reached in two cohorts	Risk of bias; imprecision due to small cohorts and limited follow-up; indirectness in specialized populations	⊕◯◯◯ Very low
CRS (any grade)	5	Frequently reported, with incidence differing across cohorts and prophylaxis subgroups	Risk of bias in retrospective ascertainment; imprecision; heterogeneity in baseline risk, prophylaxis, and grading	⊕◯◯◯ Very low
ICANS (any grade)	5	Incidence ranged from 0% to 47.6% across reported cohorts	Risk of bias in neurologic assessment; imprecision; indirectness related to differing CNS disease burden and monitoring practices	⊕◯◯◯ Very low
Severe CRS/ICANS (grade ≥ 3)	Not consistently reported	Severe CRS and ICANS were reported in selected cohorts, but outcome-specific reporting was inconsistent, preventing reliable cross-study comparisons.	Serious imprecision, inconsistent reporting, and limited event counts	⊕◯◯◯ Very low

GRADE key: ⊕◯◯◯ = very low certainty. Ratings concern confidence in descriptive outcome estimates, not comparative treatment effects. No quantitative pooling was performed.

## Data Availability

The original contributions presented in this study are included in the article. Further inquiries can be directed to the corresponding author(s).
